# Digital inclusive finance & the high-quality agricultural development: Prevalence of regional heterogeneity in rural China

**DOI:** 10.1371/journal.pone.0281023

**Published:** 2023-03-27

**Authors:** Hanjin Li, Yang Shi, Jianxin Zhang, Zhenkun Zhang, Zhaosen Zhang, Maogang Gong

**Affiliations:** College of Economics, Shandong University of Technology, Shandong, China; University of Sargodha, PAKISTAN

## Abstract

Developing digital inclusive finance is one of the most effective ways to alleviate financial exclusion in the agriculture sector. For empirical investigation, data from 30 provinces of Rural China is collected from the period 2011 to 2020. The study constructs five dimensions and 22 indicators in total to critically conduct the impact of digital inclusive finance on high-quality agricultural development. The level of agricultural development is measured by entropy weight TOPSIS, and the impact of digital inclusive finance on its high-quality development is empirically tested. The results show that digital inclusive finance has significantly improved the agricultural sector and, particularly, the Eastern region of China has the greatest impact. Three dimensions of digital inclusion finance have regional heterogeneity in terms of impact on agricultural development in Rural China. Data does not show the simple linear relationship between digital inclusion finance and agricultural development quality. The impact of the former on the latter is characterized by the double thresholds. The digital inclusive finance index is the weakest when it is lower than the first threshold that is 4.7704, and the impact of the second threshold that is 5.3186 on high-quality agricultural development is gradually enhanced. After crossing the second threshold, the impact of digital inclusive finance on high-quality agricultural development in Rural China is significantly enhanced. The development of digital inclusive finance should be strengthened in the Central and Western regions to compensate for regional financial imbalances and promote synergy in the high-quality development of agriculture across the country.

## Introduction

Since the agricultural reforms and extension, China’s agricultural economy has achieved leapfrog development under favorable production environment and efficiency. For instance, grain production has raised from 310 million tons in 1978 to 670 million tons in 2020, an increase of 2.2 times [1], and the output of agro related products such as meat, eggs, vegetables and fish have steadily ranked first in the world. Although China’s overall agricultural production capacity has improved significantly in the last few decades, but it is still far away from becoming a world’s agricultural power. Domestically, China’s agricultural production faces problems such as environmental constraints, declining arable land, lagging technological progress, slow upgrading of industrial structures and lack of human resource accumulation [2, 3]. On the other hand, developed countries have taken the lead in achieving a higher degree of agricultural modernization through advanced agricultural production technologies and government subsidies, which have put the agricultural competitiveness of developing countries far behind [4]. Therefore, if China wants to achieve stable agricultural development and resist international risks in a competitive international environment, it must explore high-quality agricultural development methods in order to achieve the goal of being a modern agricultural power.

Many scholars have conducted different empirical studies on the development of high-quality agriculture, and linked it with the stimulus factors of rural revitalization. High-quality agricultural development played a key role in the process of rural revitalization particularly in case of China. It’s also one of the major factors through which the agricultural economy can be developed and revolutionized. In a report, the former ministry of agriculture of China (2018) indicated that agriculture sector of China has been entered into the high-quality development stage which required changes in the government strategy to facilitate the agricultural stakeholders with domestic and foreign investment and banking facilities. Thus, it’s obligatory to transform the agriculture sector from ‘quantity to quality’ and “production to high productivity” [5]. There are many factors that affect quality agricultural development and it is important to allocate all factors of production efficiently; one of the key factors is finance, which helps farmers to invest in acquiring new production technologies, expanding their production operations, purchasing advanced farm equipment, introducing quality seeds, fertilizes and pesticides [6].

Rural inclusive finance has, not only, a positive role to play in counteracting external risks brought about by climate change [7], but also has an important role in narrowing the urban-rural income gap, easing credit constraints for various types of business entities, and enhancing rural human capital and agricultural technology progress [6]. However, in this digital era, traditional inclusive finance is losing its efficiency and viability in facing problems such as fragmented network institutions, restricted by time and geographical distance, single product and high application threshold [8]. On the other hand, there are multiple benefits of using Digital Inclusive Financial (DIF) services. Firstly, digital inclusion opens up new ways for farmers to access financial services and connect them to international markets. Secondly, it improves the education, health and economic status of households associated with the agricultural sector, especially the rural residents. Thirdly, it increases the per capita income and productivity of rural residents [9]. Fourthly, DIF is linked to high-quality agricultural development and the business opportunities for the rural population. This increases the rural population’s marginal propensity to consume, which leads to rural revitalization and a shift towards new production methods such as reduce waste, increase material efficiency, and renewable energy. In addition, DIF can play a positive role in reducing Sulphur Dioxide emissions and improving China’s energy and environmental performance [10, 11]. In fact, all of these develop such an ecosystem which result in the agricultural development.

**Fig 1:**
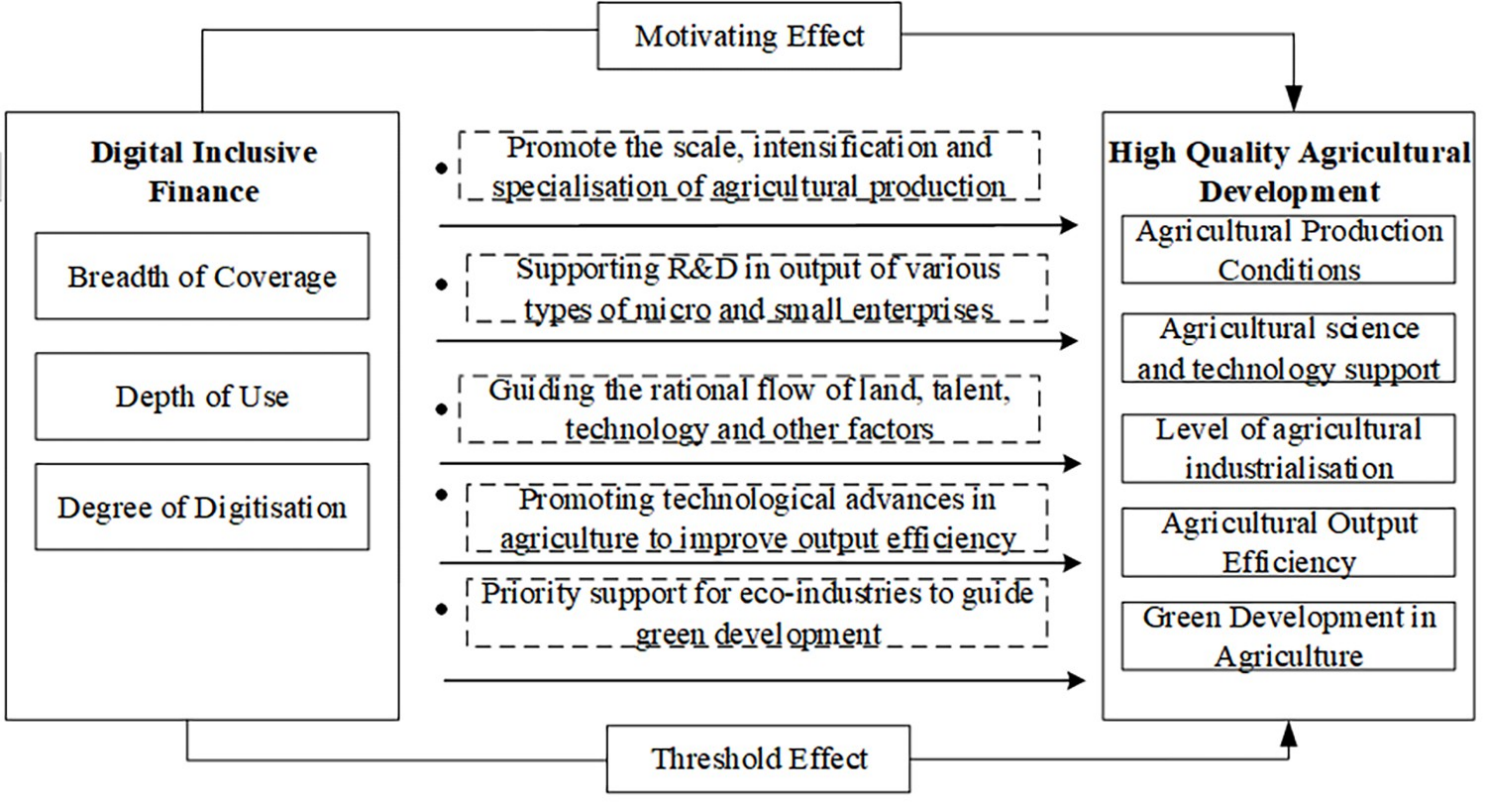
An analytical framework for digital inclusive finance impacting quality agricultural development.

Based on existing research and the realistic needs of China’s agricultural development, this paper collects data from rural areas of various provinces of China in the timespan of 2011 to 2020 in order to construct an evaluation system for its high-quality agricultural development and to empirically test the impact of DIF and its different dimensions on high-quality agricultural development. In order to further distinguish the impact of DIF on high-quality agricultural development in various regions of China, this paper also conducts heterogeneity analysis and threshold effect analysis. The analytical framework for this study is shown in Fig 1, and below questions are address in the paper:

1. How does DIF affect high-quality agricultural development in rural China?
2. How have the three components of DIF influenced high-quality agricultural development in rural China?
3. Is there regional heterogeneity in the impact of DIF on the agricultural sector in Eastern, Central and Western China?
4. Is there a threshold effect on the impact of DIF on high-quality agricultural development?

## Literature review

Agriculture has attained the pivotal position in the global economy, which not only plays a significant role in the stability of the economies but also improves the livelihood of the people, particularly, of rural areas. In this era of digitalization and information technology, agriculture has begun to adopt the technological tools to achieve convenience, high quality development, productivity and efficiency.

There are a few research works [12–14] that analyzed the impact of DIF on high-quality agricultural development, and the research outcomes, especially for the China, are worth referencing. Most of the studies inferred that DIF plays a core role in the availability of funds to rural areas and provides financial support to farmers to develop agricultural modernization and industrialization. Similarly, W. Wang et al. (2022) [9], illustrated the positive relationship between DIF and economic growth and innovative development. Although this empirical study has regional heterogeneity, but still it provides a reference for the role of inclusive finance on agricultural productivity, high-quality, and economic productivity and sustainability. The study was based upon the panel data of 31 provinces of China from 2011 to 2018.

Yu et al. [15] studied the impact of China’s inclusive finance on the development of the rural economy. It presented the argument that financial services to rural areas helped to improve the availability of funds to farmers for agricultural development. It is further studied that inclusive finance can trigger the problem of insufficient financial support for modern agricultural development caused by the unavailability of financial services, information, and limited quota. Limitations of capital supply to small farmers are behind the backwardness and unproductivity of the agricultural sector, which can be served effectively by focusing on inclusive finance in rural areas [16].

Zhang [17] conducted the study to highlight the role of DIF in promoting agricultural development. The study introduced the term of "intelligent agriculture," which is attained with access to market information, sufficient financial sources, and human resource accumulation. Zhao et al. [18] highlighted that DIF restructured agricultural activities and fostered the process of agricultural development in agricultural countries, which has narrowed the urban-rural income gap. Su & Chen [19] conducted the empirical investigation of the role of DIF in the development of the agricultural industry. The study consisted of a sample of 31 provinces of China from 2011 to 2019. The study inferred that the reduced information asymmetry, financial cost and difficulty, and educational illiteracy ensure the high-quality agricultural development, farmers’ productivity, and the establishment of agricultural industries and enterprises.

It is also studied that traditional inclusive finance has also encountered many practical problems in serving agriculture sector, rural areas and farmers. Zhu et al. [20] described that the traditional financial services to agriculture sector are characterized by small amounts, short-term financing, fragmentation, and mismatch of agricultural production cycle. Relying on the traditional inclusive financial services are not benefitting this sector for technological upgradation, high production and optimum utilization of resources. With the easy access to the internet services in rural areas, the emergence of DIF is considered to be a new way to upgrade and industrialize the agriculture sector [21–23].

In the process of supporting agricultural and rural development, DIF has introduced some innovative and result-oriented models. McIntosh & Mansini and Shamin et al. [24, 25] studied the practices of different financial supply entities in serving agricultural and rural development; these practices are divided into three types: digital financial services offered by different financial institutions, agricultural supply chain finance with agricultural leading enterprises, and financial product innovation with e-commerce and Fintech companies. To safeguard the interest of beneficiaries, here farmers and agricultural enterprises, the combination of Blockchain and traditional banking services can play a significant role for the business openness, innovation process and transparency of the financial transactions [26].

According to study of [27], the DIF developed a strong rural financial ecological environment by integrating rural commercial activities. This study empirically investigated the provinces having high rural industry integration and concluded that the DIF ultimately improved their level of mechanization in agricultural production, labour productivity, and per unit output with the integration of the financial services, industrialization and access to the international markets. In summary, it can be found that DIF has an important impact on agricultural development as it alleviates the credit constraints, optimizes industrial structure and improves agricultural productivity. Accordingly, this paper puts forward the following hypotheses.

Hypothesis 1: Digital Inclusive Finance promotes high-quality agricultural development particularly in case of Rural China.

Hypothesis 2: All three components of Digital Inclusive Finance significantly contribute high-quality agricultural development in Rural China.

Xie et al. [28] studied that the DIF has the feature of heterogeneity because it influences the agricultural development with multiple supporting factors like demography, adaptability, human capital accumulation and infrastructure. Li et al. [13] conducted the study on China and concluded that the agricultural productivity in most of its rural provinces varies because of different exogenous variables. Some of these core factors are environmental conditions, infrastructure, exposure to the availability of services and resources of each region. Accordingly, this paper puts forward the following hypotheses.

Hypothesis 3: There is the regional heterogeneity in determining the impact of Digital Inclusive Finance on high-quality agricultural development in rural areas of China.

Some scholars investigated that whether the relationship between financial development and agricultural development is a simple linear or a threshold effect. Yiting [29] highlighted that the promotion effect of financial development on agricultural development has a threshold characteristic, and this effect increases with the raise in the per capita disposable income of the rural people. Xia et al. [4] used empirical analysis from the perspective of rural economic development and farmers’ income to conclude that the role of rural financial development on rural development has time-varying characteristics. Chang [30] used the threshold effect model to illustrate the role of DIF in improving the agricultural productivity and retaining the ecosystem in rural areas of China. From various empirical studies, it is cleared that there is a threshold effect of DIF on high-quality agricultural development. Hence, we propose the following hypothesis

Hypothesis 4: The impact of Digital Inclusive Finance on high-quality agricultural development has a threshold effect.

Previous literature mainly focuses on the relationship between Inclusive Finance and agricultural development. With the increase in the accessibility of Internet technology in rural areas, studies [12, 29, 31] that empirically investigated the impact of new financial format i.e. DIF on agriculture sector. The existing literature is worth learning from, but there are still some research gaps and limitations which can be overcome through changing the estimating methods. First of all, generally the studies were conducted to analysis the impact of DIF on a single factor of agricultural development with limited scope of empirical data, particularly, in case of China. But from the perspective of agricultural high-quality development, research can be conducted more effectively if the focus is on empirical investigation of the level of agricultural development, and comprehensively measure the impact of DIF on agricultural development. Secondly, due to certain differences among the economies, histories and regions, DIF may have different levels or thresholds for high-quality agricultural development [32]. So, the existence of "digital divide" may has a negative effect on agricultural development, and it is necessary to incorporate the threshold effect in the research discussions and analyses. As single element in agricultural development cannot summarize the overall agricultural development, nor it can be effective to determine the role of DIF in influencing the overall agricultural development. This paper is intended to evaluate the status of high quality agricultural development from multiple dimensions and refer to the suitable model to analyze the impact of DIF on the level of high quality agricultural development from an empirical perspective, which to a certain extent enriches the theory of the impact of financial supply on agricultural development. In fact, it fills the research gap of the impact of DIF on the comprehensive development level of the agriculture sector.

## Indicator system, data sources and measurement methods

### Indicator system for measuring quality agricultural development

High-quality agricultural development is labelled as “AGDQ”. Subsequent table exhibits the measurement of high-quality agricultural development in the case of rural China. High-quality agricultural development contains five broad dimensions that are further classified into twenty-two (22) variables. See Table 1 for details.

**Table 1:** Indicator system for calculating high-quality agricultural development.

Calculation process of AGDQ	Contain Dimensions	Variables/Indicators	Data Sources
**High-Quality Agricultural Development**	**Comprehensive agricultural production capacity**	(1) Food production per capita in agriculture (total production/number of rural population)	China Rural Statistical Yearbook
		(2) Agricultural output rate (agricultural output value/sown area)	
		(3) Agricultural labour productivity (total output value of agriculture, forestry, animal husbandry and fishery/employees in the primary sector)	National Bureau of Statistics
		(4) Level of mechanisation of agricultural production (total mechanical power/total sown area)	China Rural Statistical Yearbook
		(5) Level of hydropower in agricultural production (effective irrigation area/total sown area)	National Bureau of Statistics
		(6) Seed technology (number of new agricultural plant variety rights authorized)	China Science and Technology Statistics Yearbook
	**Level of agricultural industrialization**	(7) Level of moderate scale operation (land transfer rate)	National Bureau of Statistics
		(8) Level of agricultural organization (total output value of agriculture, forestry, animal husbandry and fishery services/total output value of agriculture, forestry, animal husbandry and fishery)	Statistical Yearbook of Middle and Rural Areas
		(9) Structure index of farming = output value of farming industry / total output value of agriculture, forestry, animal husbandry and fishery	Statistical Yearbook of Middle and Rural Areas
		(10) Level of agricultural diversification [100 - (area sown with grain / area sown with crops)	Statistical Yearbook of Middle and Rural Areas
	**Agricultural output efficiency**	(11) Total agricultural output value	China Statistical Yearbook
		(12) Export value of agricultural products	
		(13) Stability of food production (ratio of current year to previous year)	
		(14) Value added in agriculture as a percentage of total regional output	
	**Level of agricultural welfare**	(15) Level of human capital in agriculture (years of education)	Statistical Yearbook of China’s Population and Employment
		(16) Per capita disposable income of rural residents	China Rural Statistical Yearbook
		(17) Ratio of urban to rural income	
		(18) Rural Engel’s coefficient	
	**Level of agricultural green development**	(19) Consumption of plastic film per unit (volume/sown area)	
		(20) Unit of chemical fertilizer use (amount/sown area)	
		(21) Pesticide use per unit (amount/sown area)	
		(22) Amount of green food (/)	China Green Food Development Center

Source: Author’s own calculation

The five dimensions selected for this study are described here. The conditions of agricultural production are directly related to crop yields, which are central to the measurement of agricultural development. Agricultural science and technology support can reflect to some extent the technological progress of agriculture, which is an indispensable support part of the agricultural modernization process. The level of agricultural industrialization represents the rationality of the agricultural industrial structure, and the more rational the industrial structure, the higher the level of agricultural development. Agricultural output efficiency is an important indicator of the quality development of agriculture, and the higher the output efficiency, the higher the level of agricultural development to a large extent. The level of green development in agriculture reflects the health of the agricultural economy and is an important indicator of agricultural sustainable development in the pursue of high-quality development [33].

### Measurement methods

This paper adopted a comprehensive evaluation approach to measure the high-quality development of Chinese agriculture sector. This paper selected the period 2011–2020 in which the true spirit of DIF and development period was observed in rural China. Data sources and description of indicators are given in Table 1. The entropy-weighted TOPSIS method can effectively avoid the influence of subjective factors in the process of assigning indicators, and has the advantages of simple calculation process and reasonable measurement output of both the entropy-weighted and TOPSIS methods [33]. Therefore, in order to objectively and reasonably estimate the level of high-quality development of agriculture in each province, this paper adopted the entropy-weighted TOPSIS method for measurement and evaluation, with the following equations:

(1) Data standardization processing

$$x\prime_{ij}=\frac{x_{ij}-\min(x_{j})}{\max(x_{j})-\min(x_{j})}\;(Positive\;indicator)$$

$$x\prime_{ij}=\frac{\max(x_{j})-x_{ij}}{\max(x_{j})-\min(x_{j})}\;(Negative\;indicator)$$

(2) Calculate the proportion P of index J in the ith year

$$P_{ij}=\frac{x\prime_{ij}}{\sum_{\alpha }\sum_{i}x\prime_{ij}}$$

(3) Calculate the information entropy of index J

$$e_{j}=-k\sum_{i}\;P_{ij}\;\ln(P_{ij}),Among\;k=\frac{1}{\ln(mn)},And\;k>0,\;Let\;e_{j}\geq 0$$

(4) Calculate the redundancy of the information entropy of the jth index

$$d_{j}=1-e_{j}$$

(5) Calculate the weight of index J

$$\omega_{j}=\frac{d_{j}}{\sum_{j}d_{j}}$$

(6) Calculate weighting matrix

$$X_{ij}=x\prime_{ij}\times \omega_{j}$$

(7) Calculate Euclidean distance

$${D_{i}}^{+}=\sqrt{\sum (Z_{ij}-{Z_{j}}^{*+}{)}^{2}}$$

$${D_{i}}^{-}=\sqrt{\sum (Z_{ij}-{Z_{j}}^{*-}{)}^{2}}$$

(8) Calculate comprehensive score

$$C_{i}=\frac{D_{i}^{-}}{D_{i}^{+}+D_{i}^{-}}$$

## Materials and methods

### Model descriptions

In this research, panel data of selected 30 provinces of China is used over the time period 2011–2020 for estimating results and discussion. Geographically, these provinces are further categorized into three main regions: Eastern China, Central China, and Western China. The data is collected from various sources, including China Population & Employment Statistics Yearbook, China Statistical Yearbook, National Bureau of Statistics, and China Urban Statistical Yearbook.

The projected sample population is socially deprived people belong to the rural areas of China. In today’s globalized world, mainly rural population has limited exposure to the modern technology and resources. Similarly, as habitually, rural masses rely greatly on traditional inclusive finance, which has its own drawbacks, limitations and inefficiencies. Hence, there is a need to introduce DIF in such areas to promote digitalization, uplift high-quality agricultural development and rural revitalization. For empirical evidence, researchers used two-stage least squares (2SLS), robustness regression and threshold modeling. Primarily, this research has been grounded in the following model:

$${AGDQ}_{it}=\beta_{0}+\beta_{1}{LDFI}_{it}-\beta_{2}{CPI}_{it}+\beta_{3}{LECNDQ}_{it}-\beta_{4}{LINDST}_{it}+\beta_{5}{RHMC}_{it}-\beta_{6}{URB}_{it}-\beta_{7}{GOVT}_{it}-\beta_{8}{DOP}_{it}-\beta_{9}{LDI}_{it}+\varepsilon_{it}$$ (1)

This model focuses mainly on LDIF and high-quality agricultural development (AGDQ). However, this research utilizes some control variables which are LCPI, LECNDQ, LINDS, RHMC, URB, GOVT, LDOP, and LDI. The observed DIF index is built on three major components: LBRC, LDDIG, and LDUSE. Therefore, it is necessary to incorporate the impact of these three components separately. Hence, in the 2^nd^ model of this research, the impact of LBRC on AGDQ has been examined. This model is given below.


$${AGDQ}_{it}=\beta_{0}+\beta_{1}{LBRC}_{it}-\beta_{2}{CPI}_{it}+\beta_{3}{LECNDQ}_{it}-\beta_{4}{LINDST}_{it}+\beta_{5}{RHMC}_{it}-\beta_{6}{URB}_{it}-\beta_{7}{GOVT}_{it}-\beta_{8}{DOP}_{it}-\beta_{9}{LDI}_{it}+\varepsilon_{it}$$ (2)

In the third model, researchers examined the degree of digitalization (LDDIG) and high-quality agricultural development (AGDQ). This model is as follows:

$${AGDQ}_{it}=\beta_{0}+\beta_{1}{LDDIG}_{it}-\beta_{2}{CPI}_{it}+\beta_{3}{LECNDQ}_{it}-\beta_{4}{LINDST}_{it}+\beta_{5}{RHMC}_{it}-\beta_{6}{URB}_{it}-\beta_{7}{GOVT}_{it}-\beta_{8}{DOP}_{it}-\beta_{9}{LDI}_{it}+\varepsilon_{it}$$ (3)

Furthermore, in the last and 4^th^ econometric model, the examiner considered the impact of LDDIG on AGDQ.

This model is given below.


$${AGDQ}_{it}=\beta_{0}+\beta_{1}{LDUSE}_{it}-\beta_{2}{CPI}_{it}+\beta_{3}{LECNDQ}_{it}-\beta_{4}{LINDST}_{it}+\beta_{5}{RHMC}_{it}-\beta_{6}{URB}_{it}-\beta_{7}{GOVT}_{it}-\beta_{8}{DOP}_{it}-\beta_{9}{LDI}_{it}+\varepsilon_{it}$$ (4)

The abbreviated terms occupied above displays;

AGDQ = high-quality agricultural development

LDIF = Log of digital inclusive finance index

LBRC = Log of breadth of coverage

LDDIG = Log of degree of digitalization

LDUSE = Log of depth of use

LCPI = Inflation

LECNDQ = log of economic development level (gross regional per-capita income)

LINDS = Log of industrial structure

URB = level of Urbanization (urban population/resident population)

RHMC = rural human capital accumulation

GOVT = govt. intervention (expenses on agri., forestry, & water affairs/regional GDP)

LDOP = Log of Degree of regional openness

LDI = Log of Disposable income

ε = Error term

Moreover, β_0_ reflects constant or intercept term. However, β_0_, β_2_, β_3_, β_4_, β_5_, β_6_, β_7_, β_8_, β_9_ specifies coefficients which determine the impact of explanatory variables over response variable. Here, “t” reflects the time frame over the period i.e. 2011–2020 while, “i” term denotes cross sections or the selected 30 provinces of China.

Digital Inclusion Finance (LDIF) Index has been published by Peking University’s Digital Finance Research Centre that examines nine phases of DIF that are coverage, payments, insurance, depth of use, money funds, credit, investment, credit research, and digitalization. This index is the most illustrative indicator of the extent of DIF development in rural China. It is also extensively utilized by Chinese researchers. In this research, data is collected based on three broad dimensions: breadth of coverage, the depth of usage, and the degree of digitalization. 30 provinces of China are selected because of the availability of data through reliable and credible sources.

### Descriptive statistics

Below tables shows the statistical description of the dataset. The advantage of such statistics is to summarize the all observations and present these in an organized way. The key benefit of descriptive statistics is to highlight salient features of the examined data used for the estimation and analysis. Primarily, descriptive statistics include average mean tendencies, maximum and minimum values, standard deviation, skewness, kurtosis, and the actual number of observations [34]. In order to develop descriptive statistics, Eviews-9 software has been used.

Below Table 2 shows the descriptive statistics of the dataset:

**Table 2:** Descriptive statistics.

Statistics	Mean	Max.	Min.	Std. Dev.	Skewness	Kurtosis	Prob.	Obs.
**AGDQ**	0.4007	0.5884	0.2709	0.0549	0.5357	3.3588	0.0003	300
**LDIF**	5.2192	6.0682	2.9085	0.6683	-1.5632	5.0347	0.0000	300
**LBRC**	5.0747	5.9839	0.6729	0.8201	-2.1251	8.8181	0.0000	300
**LDDIG**	5.5103	6.1360	2.0255	0.6975	-2.0328	7.0158	0.0000	300
**LDUSE**	5.2009	6.1917	1.9110	0.6475	-1.6245	6.5654	0.0000	300
**LCPI**	4.6298	4.6666	4.6108	0.0113	1.4788	4.8646	0.0000	300
**LECND**	10.8406	12.0130	9.7058	0.4361	0.3622	2.8425	0.0322	300
**LINDS**	3.8730	4.4296	3.4843	0.1733	0.7327	4.6208	0.0000	300
**RHMC**	8.0595	10.021	6.5997	0.5268	0.4224	4.8262	0.0000	300
**URB**	0.5880	0.9376	0.3503	0.1242	0.8707	3.4515	0.0000	300
**GOVT**	0.2648	2.1037	0.0807	0.3124	3.8516	19.1159	0.0000	300
**LDOP**	0.2742	1.4637	0.0075	0.2898	1.8923	6.1995	0.0000	300
**LDI**	9.3612	11.634	8.2711	0.4380	0.4735	4.8206	0.0000	300

Source: Author’s calculations using Eviews

The largest mean value is of LECND which is 10.8406. Additionally, the largest maximum value is also of LECND, which is approximately 12.0130 units, while the smallest/minimum value is of LDOP, which is approximately 0.0075. Furthermore, as compared to other variables, LBRC possesses the highest standard deviation, which is 0.8201, while the lowest standard deviation is that of inflation (LCPI), which is 0.0113. Here, LDIF, LBRC, LDDIG, and LDUSE are negatively skewed. On the other hand, LCPI, LECND, LINDS, RHMC, URB, GOVT, LDOP, and LDI are positively skewed. As far as normality of data is concerned, all variables are normally distributed towards their respective mean values. In addition, all the research variables in this research are leptokurtic. In the last column of descriptive statistics, the actual number of observations has been displayed, which is 300.

After checking prominent characteristics of the dataset, the next step is to evaluate the inter-correlation between considered variables. For this purpose, correlation matrix is used which is used to determine the degree and nature of association between the variables under consideration. Furthermore, with the help of this, one can easily detect the multi-coordinate presence [35]. In below Table 3, the correlation matrix is shown;

**Table 3:** Correlation matrix.

Variables	AGDQ	LDIF	LBRC	LDDIG	LDUSE	LCPI	LECND	LINDS	RHMC	URB	GOVT	LDOP	LDI
**AGDQ**	**1**	0.5315	0.5171	0.4412	0.5287	-0.2708	0.4396	0.2198	0.3662	0.2802	-0.0101	0.0164	0.5815
**LDIF**	0.5315	**1**	0.8764	0.8931	0.8617	-0.7434	0.5516	0.5465	0.3416	0.4212	0.2018	0.0745	0.7223
**LBRC**	0.5171	0.8764	**1**	0.8185	0.8369	-0.7100	0.5759	0.5276	0.3716	0.4546	0.1926	0.1199	0.7211
**LDDIG**	0.4412	0.8931	0.8185	**1**	0.8028	-0.7878	0.3215	0.3797	0.1755	0.1848	0.1233	-0.1674	0.4930
**LDUSE**	0.5287	0.8617	0.8369	0.8028	**1**	-0.6754	0.5699	0.5382	0.3751	0.4415	0.1951	0.1344	0.7532
**LCPI**	-0.2708	-0.7434	-0.7100	-0.7878	-0.6754	**1**	-0.2208	-0.2328	-0.1019	-0.1389	-0.0646	0.1106	-0.3603
**LECND**	0.4396	0.5516	0.5759	0.3215	0.5699	-0.2208	**1**	0.6234	0.6270	0.8899	0.4570	0.6512	0.8487
**LINDS**	0.2198	0.5465	0.5276	0.3797	0.5382	-0.2328	0.6234	**1**	0.4927	0.6737	0.6728	0.5398	0.6437
**RHMC**	0.3662	0.3416	0.3716	0.1755	0.3751	-0.1019	0.6270	0.4927	**1**	0.6651	0.4141	0.5071	0.5942
**URB**	0.2802	0.4212	0.4546	0.1848	0.4415	-0.1389	0.8899	0.6737	0.6651	**1**	0.5146	0.7655	0.7642
**GOVT**	-0.0101	0.2018	0.1926	0.1233	0.1951	-0.0646	0.4570	0.6728	0.4141	0.5146	**1**	0.4346	0.3779
**LDOP**	0.0164	0.0745	0.1199	-0.1674	0.1344	0.1106	0.6512	0.5398	0.5071	0.7655	0.4346	**1**	0.4957
**LDI**	0.5815	0.7223	0.7211	0.4930	0.7532	-0.3603	0.8487	0.6437	0.5942	0.7642	0.3779	0.4957	**1**

Source: Authors calculations using Eviews

Table-3 exhibits the correlation between the dependent and independent variables of this research work. Each variable has a perfect correlation with itself, as demonstrated by diagonal number 1. Consequently, correlation is confirmed with other variables ranges between 0 and 1. Here, no variable possesses the issue of multi-colinearity or high correlation with other variables. High-quality agricultural development (AGDQ) has an inveterate positive correlation with all variables, although AGDQ has a negative effect on LCPI and GOVT variables. Additionally, LDIF, LBRC, LDDIG, and LDUSE have positive correlation with all other variables except LCPI. Nonetheless, LCPI has negative influence on all variables except LDOP. However, LECND, LINDS, RHMC, and URB cause positive effects on all variables except LCPI. Furthermore, GOVT has a negative effect on AGDQ and LCPI while having a positive effect on LDIF, LBRC, LDDIG, LDUSE, LECND, RHMC, URB, LDOP, and LDI. Besides, LDOP exerts a positive influence on all variables of this study except LDDIG. In the end, LDI has positive correlation with all variables except LCPI.

In line with the correlation matrix and with the help of causality plotting, cause and effect association between two examined series can be determined. For this purpose, Granger causality is used to determine the causality among the research variables via Eviews software. This estimation can replicate that either the correlation between the variables is bidirectional or linear in nature. In its most basic form, it demonstrates that either ’x’ factor causes ’y’ or ’y’ also causes ’x’. If only ‘x’ causes an effect on the ‘y’ factor while ‘y’ does not cause an effect on ’x’, then it can be inferred that the nature of association is linear. But if both factors cause an effect on each other, the association is said bidirectional. A Granger causality table is presented in below Table 4;

**Table 4:** Granger causality test.

Null Hypothesis:	Obs	F-Statistic	Prob.	Conclusion
LDIF does not Granger Cause AGDQ	300	2.47187	0.0866	LDIF AGDQ
AGDQ does not Granger Cause LDIF		1.57168	0.0299	
LBRC does not Granger Cause AGDQ	300	0.40752	0.6658	LBRC AGDQ
AGDQ does not Granger Cause LBRC		3.01702	0.0508	
LDDIG does not Granger Cause AGDQ	300	0.11777	0.8890	UDDIG AGDQ
AGDQ does not Granger Cause LDDIG		10.4809	0.3005	
LDUSE does not Granger Cause AGDQ	300	3.63622	0.0278	LDUSE AGDQ
AGDQ does not Granger Cause LDUSE		5.13740	0.0066	
LINDS does not Granger Cause AGDQ	300	2.27830	0.1047	LINDS AGDQ
AGDQ does not Granger Cause LINDS		0.79285	0.4538	
LCPI does not Granger Cause AGDVQ	300	6.16638	0.0025	LCPI AGDQ
AGDVQ does not Granger Cause LCPI		3.54363	0.0305	
LECND does not Granger Cause AGDQ	300	4.09602	0.0178	LECND AGDQ
AGDQ does not Granger Cause LECND		2.55945	0.0795	
RHMC does not Granger Cause AGDQ	300	0.22390	0.0996	RHMC AGDQ
AGDQ does not Granger Cause RHMC		0.11957	0.8874	
URB does not Granger Cause AGDQ	300	0.12152	0.8856	URB AGDQ
AGDQ does not Granger Cause URB		0.87426	0.4185	
GOVT does not Granger Cause AGDQ	300	1.91259	0.1500	GOVT AGDQ
AGDQ does not Granger Cause GOVT		0.33880	0.7130	
LDOP does not Granger Cause AGDQ	300	3.63277	0.0279	LDOP AGDQ
AGDQ does not Granger Cause LDOP		2.93703	0.0550	
LDI does not Granger Cause AGDQ	300	9.31834	0.0001	LDI AGDQ
AGDQ does not Granger Cause LDI		2.82506	0.0613	

Source: Author’s calculations using Eviews

It is estimated that LDIF and AGDQ have two-ways static effect on each other. Therefore, it is confirmed that there exists a bidirectional relationship between these two variables and this effect is statistically significant. it is also estimated that the LBRC does not cause an effect on AGDQ, although AGDQ causes a static influence on LBRC; therefore, the change is linear in nature here. In case of LDDIG and AGDQ, there’s no cause and effect relationship as LDDIG fails to reject the null hypothesis, which states that LDDIG does not cause an effect on AGDQ and AGDQ does not cause an effect on LDDIG. Besides, LDUSE has two-way causality relationship with AGDQ as the null hypothesis is rejected which states that LDUSE does not cause AGDQ and vice versa. For LINDS and AGDQ, there’s no cause and effect relationship between them, as LINDS fails to reject the null hypothesis. Nonetheless, LCPI confirms bidirectional association with AGDQ, and LECND also confirms bidirectional association with AGDQ. For RHMC, there exists a linear relationship. RHMC determines AGDQ, but AGDQ does not determine RHMC. In the case of URB and GOVT, both do not have cause an effect on AGDQ; hence, it is shown that there’s no bidirectional relationship. Furthermore, LDOP asserts cause and effect association with AGDQ, and AGDQ also asserts cause and effect association with LDOP. In the end, LDI has two-way causality with AGDQ.

After analyzing the cause and effect relationship among the variables, the next step is to check the stationarity of the data set for which unit root test is used. Basically, stationary test is performed to detect the presence of unit root in the observations. A data is said to be stationary, only if its mean, variance, and the covariance values remain constant over the flow of time [36]. Below Table 5 shows the result of unit root test;

**Table 5:** Unit root test (using the LLC test and IPS test).

Variable	Differential pre-series	Stability		First order post-differential sequence	Stability
	LLC test	IPS test		LLC test	
**AGDQ**	0.5914 (0.7229)	5.2557 (0.0000)	**Stable**	-9.9424*** (0.0000)	**Stable**
**LDIF**	6.9972	0.1967	**Stable**	-31.9625*** (0.0000)	**Stable**
	(0.0000)	(0.5780)			
***LBRC***	3.6005	-1.3763***	**Stable**	0.9696	**Unstable**
	(0.9998)	(0.0084)		(0.8339)	
***LDDIG***	-10.3338*	-8.4953***	**Stable**	-15.6993***	**Stable**
	(0.0000)	(0.0000)		(0.0000)	
***LDUSE***	3.5395	2.6161	**Unstable**	-23.2029***	**Stable**
	(0.9998)	(0.9956)		(0.0000)	
***LCPI***	4.2219*	-0.2844	**Stable**	0.7423*** (0.0071)	**Stable**
	(0.0000)	(0.3881)			
***LECNDVQ***	-3.6652*	1.8348	**Stable**	0.9882	**Unstable**
	(0.0001)	(0.9667)		(0.8385)	
***LINDS***	-10.6884*	-1.1667	**Stable**	-4.9960*** (0.0000)	**Stable**
	(0.0000)	(0.1217)			
***RHMC***	-1.9069*	2.3319	**Stable**	-4.0390***	**Stable**
	(0.0183)	(0.9901)		(0.0000)	
***URB***	-5.4665*	4.2195	**Stable**	-1.0212	**Unstable**
	(0.0000)	(1.0000)		(0.8464)	
***GOVT***	-2.3866* (0.0085)	1.6152 (0.9469)	**Stable**	-7.7391*** (0.0000)	**Stable**
***LDOP***	-4.6913* (0.0000)	0.1349 (0.5537)	**Stable**	-8.1844*** (0.0000)	**Stable**
***LDI***	-9.2960* (0.0000)	-1.0118*** (0.1558)	**Stable**	-7.7110*** (0.0000)	**Stable**

*Source*: *Author’s calculations using Eviews* ***Note*:** *prob*. *Value is in ()*. And “***, **, *” shows significance at 1%, 5%, and 10% level.

Here, ‘*Common Unit Root’* (presented by Levin, lin, and Chu) and ‘*Individual Unit Root*’ (by Im, Pesaran & Shin) tests are used to check the stationarity of the dataset. Here, two grounds are considered: *‘level’* (differential pre-series) and the *‘first difference*’ (post differential series) to determine the data stationarity. All employed variables of this study affirmed their significance at 1%, and there is no presence of unit root in our dataset. AGDQ, LDIF, LDDIG, LDUSE, LCPI, LINDS, RHMC, GOVT, LDOP, and LDI confirmed their significance at 1% at selected criteria of 1st difference. Though, LBRC, LECNDQ, and URB are significant at level. Except LDUSE, all other variables such as AGDQ, LDIF, LDDIG, LDUSE, LCPI, LINDS, RHMC, GOVT, LDOP, and LDI are significant at 1% pre-differential series. In other words, except LDUSE all other research variables are integrated of order I (0).

Endogenous problems can lead to biased estimation parameters of fixed effect models. In order to diagnose whether there are endogenous problems caused by incorrect model designation, measurement error, missing variables or simultaneity, the Hausman test is used. The Hausman test is also called "Durbin Wu Hausman" (DWH) and endogenous "enhanced regression" test. See below Table 6 for complete results of the Hausman test;

**Table 6:** Hausman test.

Models	Chi^2^	Prob>Chi^2^
Model-1: ***LDIF***	15.5945	0.0758
Model-2: ***LBRC***	17.5855	0.0403
Model-3: ***LDDIG***	15.6553	0.0744
Model-3: ***LDUSE***	20.3157	0.0161

Source: Author’s calculations using Eviews

It is estimated that the P values of all variables of Hausman test are less than 0.1; therefore, there is no issue of endogenous in the model. When there is no endogenous problem, we can choose OLS for regression analysis or Two-Stage Least Square (2SLS) which is mostly applied for structural equations and an extension of simple OLS test. Here, 2SLS is used when error terms of dependent variable correlates with the independent variable. Furthermore, it examines the ‘Local Average Treatment Effect’ (LATE) whereas simple OLS determines the ‘Average Treatment Effect’ (ATE). Hence, it is better to apply 2SLS as it has the same assumptions of OLS regression which are: observations needs to be independent, error term variance for all variables must be the same, error term needs to be normally distributed, and the absence of outliers. The following Table 7 exhibits 2SLS estimations of all four models discussed in this research work.

**Table 7:** Results of 2SLS estimation of the impact of digital inclusion finance on high-quality agricultural development.

Variable	Model-1 (LDIF)	Model-2 (LBRC)	Model-3 (LDDIG)	Model-4 (LDUSE)
**LDIF**	0.6137**	-----	-----	-----
	(0.0090)			
	[0.0268]			
**LBRC**	-----	0.3058**	-----	-----
		(0.0062)		
		[0.0488]		
**LDDIG**	-----	-----	0.2116*	-----
			(0.0069)	
			[0.0931]	
**LDUSE**	-----	-----	-----	0.3372**
				(0.0079)
				[0.0398]
**LCPI**	-0.5644*	-0.3801	-0.5942*	-0.2744
	(0.3349)	(0.3056)	(0.3315)	(0.2899)
	[0.0930]	[0.2146]	[0.0741]	[0.3447]
**LECND**	0.0357***	0.0375***	0.0362	0.0393***
	(0.0143)	(0.0143)	(0.0142)	(0.0141)
	[0.0133]	[0.0092]	[0.0113]	[0.0059]
**LINDS**	-0.0124	-0.0017	-0.0085***	0.0023
	(0.0255)	(0.0241)	(0.0240)	(0.0241)
	[0.6270]	[0.9415]	[0.7227]	[0.9233]
**RHMC**	0.0234***	0.0233***	0.0234***	0.0235***
	(0.0059)	(0.0059)	(0.0058)	(0.0059)
	[0.0001]	[0.0001]	[0.0001]	[0.0001]
**URB**	-0.1275***	-0.1339***	-0.1279***	-0.1330***
	(0.0507)	(0.0506)	(0.0506)	(0.0512)
	[0.0126]	[0.0086]	[0.0121]	[0.0099]
**GOVT**	-0.0324***	-0.0343***	-0.0350***	-0.0357***
	(0.0105)	(0.0105)	(0.0102)	(0.0105)
	[0.0024]	[0.0013]	[0.0007]	[0.0008]
**LDOP**	-0.0516***	-0.0562***	-0.0486***	-0.0585***
	(0.0141)	(0.0137)	(0.0146)	(0.0134)
	[0.0003]	[0.0001]	[0.0010]	[0.0000]
**LDI**	0.0726***	0.0774***	0.0773***	0.0784***
	(0.0125)	(0.0119)	(0.0114)	(0.0129)
	[0.0000]	[0.0000]	[0.0000]	[0.0000]
**C**	-3.3948**	-2.5987**	-3.5896**	-2.1448
	(1.5232)	(1.4022)	(1.5366)	(1.3367)
	[0.0266]	[0.0649]	[0.0202]	[0.1097]
**R** ^**2**^	0.8090	0.7865	0.7908	0.7554
**F-Statistics**	33.4109***	33.0822***	33.5219***	32.9338***
	[0.0000]	[0.0000]	[0.0000]	[0.0000]

*Source*: *Author’s calculations using Eviews* ***Note*:** robust standard errors are in () and in the [] is the prob. value. And “***, **, *” shows significance at 1%, 5%, and 10% level.

Models I to IV consider the impact of the total DIF Index, and its breadth of coverage, digitization and depth of use respectively, on high quality development in agriculture. As shown in Table 6, the overall regression results of the model are good with all variables having a significant positive impact on high-quality agricultural development. Among the control variables, rural human capital accumulation as well as disposable income per capita have positive impact on high quality agricultural development. The higher the level of rural human capital accumulation, the stronger its spillover effect, and the more highly intelligent and qualified personnel can play significant role for high-quality agricultural development particularly in case of China. The higher the per capita disposable income in rural areas, the easier it is for farmers to move from the pursuit of "quantity" to the pursuit of "quality".

The estimated results, as given above, can be justifiable with strong arguments. First, efficient rural credit markets and improved banking infrastructure can promote agricultural growth [7]. The development of DIF in rural China can improve the accessibility of financial services. Through advanced digital technology, DIF breaks the spatial and temporal limitations of the traditional financial services and, as a result, more agricultural operators and participants in agricultural development enjoy rural financial services which leads to alleviating the problem of budget constraints caused by insufficient funds and ultimately promote the high-quality agricultural development in many ways. Secondly, under the ecosystem of DIF, living standard of rural areas improves and poverty alleviates. They utilize the financial and non-financial resources in a better way with the easy access to the more business opportunities and markets [12]. Finally, DIF can simultaneously enhance the degree of infrastructure, which provides better basic conditions for the development of digital and technological agriculture, and to a certain extent promotes the agricultural enterprises. Based on the above analysis, H_01_ is accepted that is DIF can promote high-quality agricultural development.

Although economic development improvement (LECND) will have an affirmative effect on high-quality agricultural development (AGDQ), there’s a 0.03 change in AGDQ from LECND. Moreover, increase in per-capita level improves the living standards of the rural people, which is one of the driving factors for high-quality agricultural development. However, LINDS asserts a negative effect on AGDQ; there’s a 0.01 decline in AGDQ because of LINDS. Improving industrial structure while ignoring agriculture will undoubtedly impede high-quality agricultural development. Besides, RHMC causes a 0.05 increase in AGDQ. The reason behind this affirmative association is that advancement in rural human capital (an increase in the number of educated and skilled labors) stimulates high-quality agricultural development [16]. Additionally, URB has negative relationship with AGDQ; there’s a 0.12 decline in AGDQ from one unit increase in the URB. An increased level of urbanization results in less facilitated rural areas, which has a negative influence on high-quality agricultural development. Similarly, GOVT causes a 0.03 decline in AGDQ which means that increased government intervention also hinders the path of high-quality agricultural development. LDOP also asserts a negative effect on AGDQ; it causes a 0.5 decline in AGDQ. LDI has a positive relationship with high-quality agricultural development; an increase in the personal incomes of the rural people will improve their living standards, education level, and agricultural technology adoption rate, which in turn raises the chances of attaining high-quality agricultural development [18, 37]. At t he end, the R-square value is 0.80, which asserts that the examined model is quite good and well explained.

The results from Models 2, 3 and 4 show that all three dimensions of LDIF contribute positively to AGDQ. Among them, the LDUSE leads to 33.72% change in AGDQ, which has the highest impact among the selected dependent variables; the LBRC has the second highest degree of impact on AGDQ which is about 30.58%; the LDDIG has the relatively smallest impact on AGDQ which is 21.16%. Reason for this phenomenon may be that the LDUSE consists of credit, investment, insurance and fund businesses. For high agricultural production and operational activities, the business entities and enterprises can better invest and allocate the funds through online banking system. This expands the agriculture sector through industrialization, diversification in product portfolio, access to different local and international markets and utilization of idle funds for productive activities. The LDDIG, on the other hand, is in terms of affordability and creditworthiness, suggests that there are still some shortcomings with regard to interest rate bands and credit issues in LDIF.

Despite the different degrees of impact, overall all three dimensions contribute to AGDQ, and the estimation leads to the acceptance of the hypothesis H_02_. For checking the creditability of long run results obtained from 2SLS, there is a need to assimilate the creditability and significance of the results. Hence, for this purpose, robustness regression has been applied. With the help of robustness test, it can be confirmed that the dataset has the reliability, accuracy, and creditability of Table 7 statistics. See below the Table 8 for results;

**Table 8:** Robustness tests.

Variable	Model-1 (LDIF)	Model-2 (LBRC)	Model-3 (LDDIG)	Model-4 (LDUSE)
**LDIF**	0.5102***	-----	-----	-----
	(0.0072)			
	[0.0168]			
**LBRC**	-----	0.3054**	-----	-----
		(0.0049)		
		[0.0215]		
**LDDIG**	-----	-----	0.2001*	-----
			(0.0055)	
			[0.0794]	
**LDUSE**	-----	-----	-----	0.0187***
				(0.0062)
				[0.0030]
**LCPI**	-0.1112	-0.2089	-0.3846**	-0.0261***
	(0.2685)	(0.2444)	(0.2675)	(0.2298)
	[0.6788]	[0.3925]	[0.0505]	[0.0094]
**LECND**	0.0759***	0.0763***	0.0737***	0.0711***
	(0.0114)	(0.0114)	(0.0114)	(0.0112)
	[0.0000]	[0.0000]	[0.0000]	[0.0000]
**LINDS**	0.0454***	0.0422**	0.0336*	0.0425***
	(0.0205)	(0.0192)	(0.0194)	(0.0191)
	[0.0267]	[0.0284]	[0.0836]	[0.0264]
**RHMC**	0.0277***	0.0284***	0.0280***	0.0271***
	(0.0047)	(0.0047)	(0.0047)	(0.0047)
	[0.0000]	[0.0000]	[0.0000]	[0.0000]
**URB**	-0.3857***	-0.3827***	-0.3735***	-0.3906***
	(0.0407)	(0.0405)	(0.0408)	(0.0406)
	[0.0000]	[0.0000]	[0.0000]	[0.0000]
**GOVT**	-0.0368***	-0.0367***	-0.0342***	-0.0373***
	(0.0084)	(0.0084)	(0.0082)	(0.0083)
	[0.0000]	[0.0000]	[0.0000]	[0.0000]
**LDOP**	-0.0356***	-0.0333***	-0.0305***	-0.0357***
	(0.0113)	(0.010)	(0.0118)	(0.0106)
	[0.0017]	[0.0024]	[0.0099]	[0.0008]
**LDI**	0.0991***	0.0942***	0.0889***	0.1155***
	(0.0100)	(0.0095)	(0.0092)	(0.0102)
	[0.0000]	[0.0000]	[0.0000]	[0.0000]
**C**	-1.9680	-2.3997**	-3.1359***	-1.3706
	(1.2213)	(1.1214)	(1.2398)	(1.0598)
	[0.1071]	[0.0324]	[0.0114]	[0.1959]
**R** ^**2**^	0.6801	0.6484	0.6435	0.6887

*Source*: *Author’s calculations using Eviews* ***Note*:** robust standard errors are in () and in the [] is the prob. value. And “***, **, *” shows significance at 1%, 5%, and 10% level.

In the subsequent Table 8, robustness in regression estimation has been exhibited for all four models examined in this research. Except for the case of LDUSE, we can conclude from Table 6 that the robustness statistics results are very similar to the 2SLS estimates in Table 5. Here, LDIF asserts 51% influence on AGDQ while it was 61% in Table 5. However, LBRC asserts the same effect, which is approximately 30%. Besides, LDDIG causes a 20% change according to robustness statistics, while it was 21% in 2SLS estimation. LDUSE asserts a 1% change here, but it was 33% in accordance with 2SLS estimation. As in Table 5, all variables are statistically significant here, with the exception of LCPI, which is insignificant for models 1 and 2. Consequently, R^2^ is 68%, 64%, 64%, and 68% for models-1, model-2, model-3, and model-4, respectively.

Next step is to check the hypothesis-3 which is about the regional heterogeneity. For this purpose, 2SLS approach is used for rural China (Eastern, Central, and Western provinces). For further details, see the following Table 9;

**Table 9:** Results of 2SLS estimation on the impact of digital financial inclusion on high-quality agricultural development in different regions.

Variable	Model-1 (Eastern)	Model-2 (Central)	Model-3 (Western)
**LDIF**	0.5187**	0.3547***	0.2101**
	(0.0195)	(0.0127)	(0.0085)
	[0.0388]	[0.0065]	[0.0390]
**LCPI**	-0.0303	-0.2409***	-0.0037
	(0.5859)	(0.4711)	(0.3046)
	[0.9588]	[0.0101]	[0.9902]
**LECND**	0.0676**	-0.0781***	0.1009***
	(0.0328)	(0.0178)	(0.0257)
	[0.0417]	[0.0000]	[0.0002]
**LINDS**	-0.0593	0.0870*	0.1026***
	(0.0672)	(0.0472)	(0.0365)
	[0.3790]	[0.0692]	[0.0063]
**RHMC**	0.0379**	0.0540***	0.0608***
	(0.0178)	(0.0153)	(0.0072)
	[0.0360]	[0.0007]	[0.0000]
**URB**	-0.2610***	0.4724***	-0.5999***
	(0.0827)	(0.0717)	(0.0788)
	[0.0021]	[0.0000]	[0.0000]
**GOVT**	-0.0357***	-0.6017***	0.0501**
	(0.0150)	(0.0541)	(0.0247)
	[0.0197]	[0.0000]	[0.0459]
**LDOP**	-0.0515**	-0.1341	0.0460
	(0.0220)	(0.1097)	(0.0422)
	[0.0209]	[0.2252]	[0.2795]
**LDI**	0.1325*	0.0354***	0.0722***
	(0.0370)	(0.0118)	(0.0225)
	[0.0005]	[0.0037]	[0.0019]
**C**	-1.5241	-5.9261***	-1.8512
	(2.6563)	(2.0732)	(1.3692)
	[0.5673]	[0.0054]	[0.1802]
**R** ^**2**^	0.6666	0.7765	0.8259
**F-Statistics**	15.9849***	30.8896***	42.1674***
	[0.0000]	[0.0000]	[0.0000]

*Source*: *Author’s calculations using Eviews* ***Note*:** robust standard errors are in () and in the [] is the prob. value. And “***, **, *” shows significance at 1%, 5%, and 10% level.

In the Table 9, regional heterogeneity estimation has been represented using 2SLS. Here, model-1 of this research has been tested at regional level. From above statistics, it is shown that LDIF emphasizes 51% change in Eastern region, 35% change in Central region, while 20% change in Western region. It is to be noted that the developed regions incur more significant effect on high-quality agricultural development than that of backward regions. Eastern China that is the utmost developed, dynamic, and advanced region of China has the most favorable result which constructs a stable path for high-quality agricultural development [38].

As far as backward regions are concerned, there is a need to focus on the infrastructure and digitalization of the banking and commercial services in order to equip them for attaining high-quality agricultural development. Followed by Eastern Region, there is Central China which is a developing region. Western region falls in the list of backward provinces of China and has the limited role in high quality agricultural development. About hypothesis-3, the null hypothesis can be rejected which can be inferred that there is the presence of regional heterogeneity. Consequently, R^2^ is 66%, 77%, and 82% for case 1, 2, and 3, respectively. Now, next step is to estimate the model-2 and the presence of regional heterogeneity. For this purpose, by using 2SLS approach, rural China (Eastern, Central, and Western provinces) has been tested for empirical examination. For better understanding see the following Table 10.

**Table 10:** Results of 2SLS estimation of the impact of three dimensions of DIF on high-quality agricultural development in different regions.

Variable	LBRC			LDDIG			LDUSE		
	Model-1 (Eastern)	Model-2 (Central)	Model-3 (Western)	Model-1 (Eastern)	Model-2 (Central)	Model-3 (Western)	Model-1 (Eastern)	Model-2 (Central)	Model-3 (Western)
**LBRC**	0.4162**	0.2334***	0.2021***						
	(0.0172)	(0.0111)	(0.0048)						
	[0.0481]	[0.0037]	[0.0098]						
**LDDIG**				0.4403*	0.3503***	0.2044**			
				(0.0105)	(0.0106)	(0.0072)			
				[0.0788]	[0.0015]	[0.0427]			
**LDUSE**							0.2621**	0.1103**	0.2198***
							(0.0178)	(0.0111)	(0.0063)
							[0.0472]	[0.0548]	[0.0026]
**LCPI**	-0.1024	-0.3056***	-0.1459**	-0.2686	-0.5043***	-0.3874	-0.0005	-0.4099	-0.1972***
	(0.5384)	(0.4669)	(1.2564)	(0.5767)	(0.4867)	(0.2859)	(0.5009)	(0.3780)	(0.2443)
	[0.8495]	[0.0065]	[0.0529]	[0.6423]	[0.0027]	[0.1702]	[0.9991]	[0.2815]	[0.4218]
**LECND**	0.0671**	0.0835***	0.0977**	0.0703**	0.0806***	0.0926***	0.0670**	0.0649***	0.0935***
	(0.0328)	(0.0182)	(0.0256)	(0.0329)	(0.0175)	(0.0266)	(0.0326)	(0.0178)	(0.0243)
	[0.0433]	[0.0000]	[0.0003]	[0.0348]	[0.0000]	[0.0008]	[0.0421]	[0.0005]	[0.0002]
**LINDS**	-0.0618	0.0861*	0.0959***	-0.0740	0.1204***	0.0928***	-0.0592	0.1381***	0.0959***
	(0.0665)	(0.0461)	(0.0366)	(0.0661)	(0.0396)	(0.0374)	(0.0644)	(0.0498)	(0.0346)
	[0.3542]	[0.0654]	[0.0106]	[0.2653]	[0.0032]	[0.0152]	[0.3601]	[0.0070]	[0.0070]
**RHMC**	0.0383**	0.0512***	0.0604***	0.0387**	0.0531***	0.0593***	0.0378**	0.0656***	0.0607***
	(0.0178)	(0.0154)	(0.0072)	(0.0179)	(0.0149)	(0.0072)	(0.0177)	(0.0153)	(0.0068)
	[0.0337]	[0.0014]	[0.0000]	[0.0329]	[0.0006]	[0.0000]	[0.0350]	[0.0001]	[0.0000]
**URB**	-0.2571***	0.4570***	-0.5884*	-0.2519***	0.4641***	-0.5682***	-0.2717***	0.4726***	-0.6041***
	(0.0822)	(0.0711)	(0.0784)	(0.0824)	(0.0703)	(0.0820)	(0.0828)	(0.0755)	(0.0742)
	[0.0023]	[0.0000]	[0.0000]	[0.0028]	[0.0000]	[0.0000]	[0.0014]	[0.0000]	[0.0000]
**GOVT**	-0.0363***	-0.6062***	0.0501**	-0.0316**	-0.6069***	0.0464*	-0.0355***	-0.6148***	0.0493***
	(0.0153)	(0.0535)	(0.0250)	(0.0143)	(0.0529)	(0.0249)	(0.0144)	(0.0568)	(0.0234)
	[0.0199]	[0.0000]	[0.0487]	[0.0300]	[0.0000]	[0.0656]	[0.0153]	[0.0000]	[0.0390]
**LDOP**	-0.0493**	-0.1455	0.0513**	-0.0487**	-0.1117	0.0546	-0.0516***	-0.0433	0.0498***
	(0.0213)	(0.1094)	(0.0421)	(0.0229)	(0.1029)	(0.0422)	(0.0212)	(0.1121)	(0.0399)
	[0.0229]	[0.1872]	[0.2275]	[0.0363]	[0.2808]	[0.1994]	[0.0167]	[0.7004]	[0.2152]
**LDI**	0.1322*	0.0358***	0.0661**	0.1138***	0.0392***	0.0590***	0.1425***	0.0413***	0.0939***
	(0.0370)	(0.0116)	(0.0220)	(0.0304)	(0.0112)	(0.0205)	(0.0365)	(0.0123)	(0.0220)
	[0.0005]	[0.0029]	[0.0036]	[0.0003]	[0.0008]	[0.0052]	[0.0002]	[0.0013]	[0.0001]
**C**	-1.8626	-6.1185***	-2.4688**	-2.5120	-7.2808***	-3.4974***	-1.4247	-2.4421	-0.9961***
	(2.4458)	(2.0360)	(1.2564)	(2.6707)	(2.1929)	(1.3003)	(2.3024)	(1.7079)	(1.1340)
	[0.4480]	[0.0035]	[0.0529]	[0.3490]	[0.0014]	[0.0087]	[0.5373]	[0.1566]	[0.3854]
**R2**	0.7865	0.7794	0.8238	0.7374	0.7840	0.8236	0.6713	0.7573	0.8419
**F-Statistics**	15.9761*** [0.0000]	31.4204*** [0.0000]	41.5596*** [0.0000]	15.7941*** [0.0000]	32.2702*** [0.0000]	41.5190*** [0.0000]	16.2922*** [0.0000]	27.7494*** [0.0000]	47.3427*** [0.0000]

*Source*: *Author’s calculations using Eviews* ***Note*:** robust standard errors are in () and in the [] is the prob. value. And “***, **, *” shows significance at 1%, 5%, and 10%

Table 10 presents the regression results of the regional heterogeneity of LBRC, LDDIG and LDUSE respectively. As it can be seen in Tables 9 and 10 that the regression results for LBRC and LDDIG follow the same trend as for the LDIF on AGDQ in Table 8 specifically in terms of the degree of impact. However, the result in Table 10 for the impact of LDUSE on AGDQ, the Eastern region is still the most influential region with a coefficient of 26.21%, followed by the Western region (21.98%) than the Central region (11.03%). There are two possible reasons for this phenomenon: first, the components of LDUSE, and the "inclusive" nature of DIF has a more significant effect on alleviating credit constraints in less developed regions. The Central region is better developed than that of Western region, so its role is not as strong as that of the Western region. The Eastern region, on the other hand, has been the most economically dynamic region in China, and is the best in terms of both acceptance of DIF and the basic conditions for agricultural development. Therefore, the Eastern region continues to have the highest degree of influence on AGDQ in terms of LDUSEI. Secondly, there may be a threshold effect on the impact of LDIF on AGDQ after a certain level is crossed. Here, the threshold characteristics is estimated, based on S. Wang [39] research method, and set up a panel threshold model to investigate its threshold characteristics. Since the exact number of thresholds is not known, a single threshold model is assumed first, and the panel threshold model is set up as follows.

$${RURAL}_{it}=\alpha_{0}+\alpha_{1}{LNDFI}_{it}(q_{i}\leq \gamma )+\alpha_{2}{LNDFI}_{it}(q=>\gamma )+\beta_{1}LNCPI+\beta_{2}LECND+\beta_{3}LINDS+\beta_{4}RHMC+\varepsilon_{it}$$ (5)

*q*_*i*_ represents the threshold variable, *ε*_*it*_ is the random error term, and the rest of the symbols indicate the same as above.

In threshold regression, a threshold value or the set of threshold values are used. The purpose behind setting threshold level is to distinguish between ranges of values where the behavior anticipated by model differs in some manners. Threshold regression quantifies accurately how much indication may change (such as potential biases, or the sampling variation) before endorsement changes, and what would be the recommendation for revision. Threshold regression follows the same assumptions as does the least square which are the linear relationship, independence of observations, homoscedasticity, and normal distribution. In addition, hypothesis-4 is tested for detecting threshold effects and determining the required threshold level. For this purpose, the threshold regression approach has been utilized for empirical investigation. For better understanding, see the following Table 11:

**Table 11:** Threshold effect test.

Threshold variable	Threshold nature	F statistic	P value	10% critical value	5% critical value	1% critical value
	Single threshold	178.58	0.00***	9.81	11.47	15.37
*LDIF*	Double threshold	52.66	0.03**	11.40	12.95	16.84
	Triple threshold	42.35	0.27	12.29	14.03	17.72

*Source*: *Authors calculations using Eviews* ***Note*:** “***, **, *” shows significance at 1%, 5%, and 10% level.

With the help of Table 11, the study tries to explicate the statistical estimates obtained from threshold effect testing. In this paper, the index of DIF has been taken as a threshold variable. LDIF has been tested against three threshold levels. As shown in the above table, significance has been checked at 10%, 5%, and 1% levels. As we see, a single threshold value is statistically significant at a confidence level of 1%, while a double threshold value is statistically significant at a confidence level of 5%. However, the triple threshold value did not pass the statistical significance criteria and it is insignificant. This shows that the effect of DIF does not have a simple linear relationship with high-quality agricultural development and, hence, there exist double threshold characteristics. This validates the acceptance of alternative hypothesis 4, and the rejection of the null hypothesis. Therefore, threshold regression estimation is made and given in below Table 12:

**Table 12:** Threshold regression results.

Parameters/Variables	Agricultural High-Quality Development (AGDQ)
**LDIF (LDIF(-1)≤4.7707)**	0.5540** (0.6348)[0.0322]
**LDIF (4.7707<LDIF ≤5.3186)**	0.6122** (0.5118)[0.0213]
**LDIF (LDIF>5.6442)**	0.8011* (0.4585)[0.0817]
**LCPI**	-0.4330*** (1.3889)[0.0000]
**LECND**	0.2675*** (0.0654)[0.0001]
**LINDS**	0.4857*** (0.1207)[0.0001]
**RHMC**	0.0331 (0.0277)[0.2339]
**URB**	-0.6447*** (0.2331)[0.0061]
**GOVT**	-0.2172*** (0.0478)[0.0000]
**LDOP**	0.1113 (0.0789)[0.1598]
**LDI**	0.2664*** (0.0597)[0.0000]
**C**	7.5581*** (6.6096)[0.0001]
**R2**	0.9334
**N**	300

*Source*: *Author’s calculations using Eviews* ***Note*:** robust standard errors are in () and in the [] is the prob. value. And “***, **, *” shows significance at 1%, 5%, and 10%

Above result shows that there is positive and statistically significant threshold effect of the DIF index on high-quality agricultural development. Besides, all of the variables have significantly passed the significance criteria at 1% level. The values of 4.7704, between 4.7704 and 5.3186, and higher than 5.6442 are regarded as low, medium, and high threshold levels, respectively. In particular, when LDIF is not higher than 4.7704, and one unit change in DIF causes the change of 0.5540 unit in high-quality development of agriculture. When LDIF is between 4.7704 and 5.3186, the role of DIF in promoting high-quality agricultural development is more static and significant. Here, high-quality agricultural development increases by 0.6122 units on an average. Additionally, when LDIF is higher than 5.6442, the role of DIF in promoting high-quality agricultural development is further increased, and coefficient impact reaches 0.8011 units.

The above statistics show that by improving the DIF the high-quality agricultural development significantly increased particularly in China. According to threshold regression estimation, all variables except RHMC and LDOP confirmed their statistical significance at 1% level. Here, R^2^ value is 93%, which confirms the goodness of fit or statistical creditability of the model. On the basis of Hypothesis-4, we can reject the null hypothesis and validate the presence of a threshold effect.

## Conclusions and policy implications

This study uses data on rural China from 2011–2020 and selects 22 indicators to construct a system for high-quality agricultural development. The entropy-weighted TOPSIS method was applied to comprehensively evaluate the level of high-quality development of Chinese agriculture. The relationship between the explanatory and response variables are subsequently analyzed through empirical tests. It was found that: firstly, DIF along with LBRC, LDDIG and LDUSE have significant contribute to the level of agricultural quality development. Secondly, there is regional heterogeneity in the effect of DIF on AGDQ, except of LDUSE which is the weakest in the case of Central region and similarly in case of East and West China. The strength of other two dimensions i.e. LBRC and LDDIG and the effect of LDIF on AGDQ show a decreasing trend in case of East, Central and West China. Finally, the results of the threshold effect model show that the relationship between DIF and AGDQ is not the linear one, and that the former has a double threshold characteristic on the latter. This shows that there is a causal relationship between the uneven development of high-quality agriculture across China and the uneven development of DIF.

Based on the above findings, following recommendations can be made.

1. Strengthen the development of DIF to promote high-quality agricultural development [40]. Firstly, the government should provide policy support and guide social capital injection into rural areas to put the policy dividends into practice. Secondly, infrastructure should be given priority which includes the electronic terminals, internet lines and other related infrastructure. Finally, financial institutions should actively promote and develop set-up for DIF knowledge to enhance the financial literacy of rural residents and promote their acceptance of IT-enabled financial services.
2. Focus of DIF development should be given to the less developed regions of the China to alleviate the regional imbalance and improve their role for high-quality agricultural development. Due economic opportunities, infrastructure, resource endowment and planting differences among regions have led to obvious regional heterogeneity. Support for lagging regions should be strengthened; DIF development should be promoted based on realistic conditions, and innovative DIF products should be offered to support the development of agricultural technology, talent, large-scale operations and specialized operations. Avoid the emergence of the horse-trust effect causing the differences between East and West parts of China.
3. Construct DIF development strategies in a comprehensive and multi-faceted manner, and adhere to the principle of appropriateness to promote the same in the rural areas of the China. The research results show that the impact of the level of DIF development on the high-quality development of agriculture has threshold characteristics, and when the second threshold is exceeded, the this effect is significantly enhanced. For regions where the LDIF index is below the second threshold, strategies to enhance DIF development should be formulated according to the local conditions, and for regions above the second threshold, DIF development should continue to be strengthened in order to facilitate stronger promotion of high-quality agricultural development.

### Limitations

The study has a number of limitations that could be considered for future research. Firstly, our study is based on the analysis of data measured by the Centre of Peking University. The data is collected from Ant Financial Services, which is highly recognized institute for providing reliable data but this is a single source of data collection and the study could not considered the data from the other financial institutions in China. Future empirical analysis can be conducted by collecting data on DIF related businesses from formal and informal financial institutions of China. Secondly, although the researchers have tried to measure the level of quality development of agriculture from different dimensions and indicators to measure its development, but still it is not enough and we hope that future studies can be conducted by measuring the agriculture development in more precise way. Finally, it is encouraged to analyze the regression of the three dimensions of DIF in more depth, such as how the credit, fund, investment and insurance businesses included in LDUSE affect the high quality development of agriculture, and what is the significance of the affordability and facilitation of LDDIG in this regards.

## Supporting information

S1 File (XLSX)
